# Knowledge and awareness of HPV vaccination uptake and recommendations in gulf cooperation council countries 2009–2025: a systematic review

**DOI:** 10.1186/s13690-026-01875-6

**Published:** 2026-03-13

**Authors:** Samiya Al Khaldi, Celine Tabche, Zeenah Atwan, Salman Rawaf

**Affiliations:** 1 Planning and Research Department, Directorate General of Health Services, Al Batinah North Governorate, Ministry of Health, Muscat, Oman; 2 Faculty of Medicine, WHO Collaborating Centre for Public Health Education and Training, School of Public Health, Imperial College London, 90 Wood Lane, London, W12 0BZ UK; 3 Department of Microbiology, Central Laboratory, College of Medicine, University of Basrah, Basrah, Iraq

**Keywords:** HPV, Papillomavirus vaccine, GCC, Cancer prevention, Public health, Vaccination

## Abstract

Cervical cancer, largely caused by persistent infection with human papillomavirus (HPV), remains a preventable disease through timely vaccination. However, in the Gulf Cooperation Council (GCC) countries, the uptake of the HPV vaccine has been inconsistent, and public knowledge remains limited. This systematic review aims to synthesise available evidence on HPV vaccine awareness, uptake, barriers, enablers, and recommendations in the GCC region.

**Methods** A systematic search of peer-reviewed literature was conducted across major databases to identify studies published till March 2025. Eligible studies included cross-sectional, quasi-experimental, and descriptive studies reporting on HPV-related knowledge, attitudes, vaccine uptake, barriers, enablers, and recommendations in the six GCC countries. Data were extracted and synthesised thematically. The screening process followed PRISMA guidelines, and 52 studies were included in the final analysis.

**Results** The study included 52 articles. While awareness of cervical cancer was moderate to high in several studies, knowledge of HPV infection and the HPV vaccine was generally low. Actual vaccine uptake rates were poor across all countries, with most reporting rates below 10%, except for Abu Dhabi, which reported the highest uptake (> 95%) through its school-based programme. Key barriers included lack of knowledge, safety concerns, cultural and religious beliefs, cost, and the absence of national immunisation programmes. Enablers included personal willingness, trust in healthcare providers, prior health education, and availability of free vaccination. The studies recommended urgent policy change across GCC countries to incorporate the HPV vaccine into national vaccination programmes.

**Conclusion** Despite the established link between HPV and cervical cancer, vaccine uptake remains low across the GCC due to persistent knowledge gaps, sociocultural barriers, and systemic limitations. This review highlights the urgent need for coordinated public health strategies that prioritise education, equitable access, and national policy integration.

**Supplementary Information:**

The online version contains supplementary material available at 10.1186/s13690-026-01875-6.

**Table:** 

Text box 1: Contributions to the literature
• This is the first systematic review to map knowledge, attitudes, barriers, and enablers to HPV vaccination across all six GCC countries
• It highlights striking differences in uptake, with school-based, government-funded programmes achieving high coverage while private-sector reliance limits access
• The review identifies consistent gaps in awareness of HPV and its vaccine, even among healthcare providers
• It underscores cultural, social, and structural barriers that require context-specific interventions
• It calls for integrating HPV vaccination into national immunisation schedules to align with global cervical cancer prevention efforts

## Background

Human papillomavirus (HPV) can infect females and males over a lifetime. Among women aged 50 years and older with abnormal cervical cytology, HPV was prevalent in 54.5% (95% CI, 46.0% to 62.8%), with 43.0% (95% CI, 36.6% to 49.5%) being high-risk HPV (HR-HPV) [1]. According to global estimates, HPV caused more than half of all infection-attributable cancers in women worldwide. In low human development index countries, it accounted for half of infection-attributable cancers in both sexes combined [2–4]. The virus is well connected to various types of cancers, such as cervical cancer, vagina, vulva, head and neck, anal and penile carcinomas [5]. Based on their ability to express oncogenic proteins, HPV types are divided into high-risk (HR—carcinogenic) or low-risk (LR- non-carcinogenic) [6, 7]. The 12 most common HPV types are 16, 18, 58, 33, 45, 31, 52, 35, 59, 39, 51, and 56. Globally, HPV-16 and 18 together account for approximately 70% of invasive cervical cancer (ICC) cases, with HPV-16 responsible for around 50–60% and HPV-18 for 10–15% individually. However, this percentage varies according to the geographical area; for instance, in West/Central Asia alone, the corresponding ICC cases for HPV-16 and 18 reaches 82% [8].

Besides infection with HR-HPV types, developing precancerous lesions and cancer is boosted by several factors, including multiparity, smoking, contraceptive pills, coinfections, diet, genetic susceptibility and other STD infections such as HIV and Chlamydia and multiple types of HPV infections [9].

Although HPV is associated with multiple types of cancers, cervical cancer (CC) remains the most widely recognised by the public. CC is the fourth most common diagnosed cancer and the fourth leading cause of death among women. Globally, 604,000 and 342,000 new cases and deaths were reported, respectively [10]. CC is largely preventable through comprehensive HPV vaccination programmes, particularly when vaccination is administered at young ages prior to HPV exposure; recent evidence indicates that one- or two-dose schedules can provide effective protection against high-grade cervical lesions in adolescents, although vaccine effectiveness declines substantially when vaccination is initiated at age 25 years or older, underscoring the importance of early immunisation alongside organised screening [11, 12]. The available vaccines can trigger high levels of neutralising antibodies through non-infectious virus-like particles (VLP). Since HPV 16 and 18 types are the most common oncogenic, the available vaccines Gardasil quadrivalent, Cervarix bivalent, Gardasil 9 nonvalent, and Cecolin bivalent are designed to include their VLPs in their components [13].

To date, the HPV vaccines have been implemented in national immunisation programmes in 125 countries (64%) for girls and in 47 countries (24%) for boys [2]. The WHO’s three key goals are providing vaccines to 90% of girls by the age of 15, screening cervical cancer for 70% of women aged 30–49 (using a high-performance test by the age of 35 and again by the age of 45) and providing adequate treatment for 90% of women who have been identified with pre-cancerous lesions or CC [14].

Recently, HPV prevalence has significantly increased in the Middle East [15]. Varying HPV prevalence rates were reported in the GCC region. In Saudi Arabia (SA), for example, studies showed that prevalence ranges from 15.1%−31% and 43% in different geographical locations across the kingdom [16, 17]. While another study approximated the prevalence of HR-HPV among women living in the rest of the GCC countries to be 21% [7].

However, the provision of HPV vaccines gives a great opportunity to prevent cervical cancer and other HPV-related diseases. The introduction of HPVV has been slow in the Eastern Mediterranean region because of the stigma associated with sexually transmitted infections like HPV in a conservative society [18]. In addition, the governments of GCC countries doubt the cost-effectiveness of the national HPV vaccination programme [19]. Hence, these factors hinder the implementation of HPV vaccine programmes in the GCC countries. The aim of this systematic review is to synthesise available evidence on the knowledge, attitudes, and uptake of the HPV vaccine across the GCC countries. Specifically, the review seeks to examine the level of awareness regarding HPV infection and its link to cervical cancer, assess the extent of HPV vaccine uptake, and identify the key barriers and enablers influencing vaccination decisions. Additionally, the review aims to collate recommendations provided by the included studies to inform policy development, public health programming, and future research. By consolidating findings from diverse populations and settings within the GCC region, this review provides a comprehensive understanding of the current landscape and highlights actionable strategies for improving HPV vaccine coverage and cervical cancer prevention.

## Methods

The search strategy for the systematic review plan was registered in PROSPERO under the identification code CRD420251008629 [20]. A Covidence PRISMA checklist was used to evaluate adherence to standardised methodologies for systematic reviews [21].

### Search strategy

Embase®, Medline, Global Health, Health Management Information Consortium, PubMed, APA PsycINFO, Maternity and Infant Care Database, WHO Global Index Medicus, Cochrane, Scopus, and Web of Science were searched. The search included MeSH terms and free text within each database till March 2025. Search syntax according to the listed databases is shown in Supplementary Table 1. No articles were found on Scopus, WHO GIM, and Cochrane Library of Systematic Reviews**.** No meta-analysis was performed because the trials included in this review were heterogeneous with respect to population, intervention, and comparator. The inclusion and exclusion criteria for selecting the literature are shown in Table 1.

**Table 1:** The inclusion and exclusion criteria used for screening on covidence

	Inclusion criteria	Exclusion criteria
Population	Any age and gender residing in one or more of the Gulf Cooperation Council (GCC) countries: SA, Oman, United Arab Emirates, Qatar, Kuwait, and Bahrain	Outside the GCC region or not reporting GCC-specific findings Studies focusing solely on individuals with a diagnosis of cervical cancer or other HPV-related diseases without discussing vaccine-related outcomes
Intervention	HPV infection, HPV vaccination, cervical cancer awareness, attitudes, uptake, barriers, enablers, or health beliefs related to HPV vaccination	Articles that did not focus on HPV infection, HPV vaccine, or cervical cancer prevention (e.g. focused exclusively on other sexually transmitted infections or unrelated health outcomes)
Study Design	Quantitative, qualitative, or mixed-methods studies, including cross-sectional surveys, cohort studies, descriptive studies, and quasi-experimental designs	Editorials, commentaries, letters to the editor, conference abstracts, protocols, reviews (systematic or narrative), or studies without primary data
Outcome	Awareness or knowledge of HPV, HPV-related diseases, or HPV vaccine Uptake or coverage of HPV vaccine Attitudes, perceptions, beliefs, or willingness to receive HPV vaccination Barriers or facilitators/enablers of HPV vaccine uptake Recommendations for improving vaccine uptake	Articles that lacked sufficient data on key outcomes of interest (e.g. only general statements on HPV awareness without reporting specific results) Studies that do not record any information about HPV interventions/programmers’ coverage rates, effectiveness, barriers and enablers
Language	Published in English or Arabic	Articles with full text are not found or published in English and Arabic

After removing duplicates, two authors independently screened the titles, abstracts, and full texts of articles and included eligible articles for full-text review. An independent third author resolved any disagreements. We conducted the screening and full-text review in Covidence. Only peer-reviewed records in English and Arabic were included. No limitations for the period were specified.

### Assessment of studies’ quality

The Joanna Briggs Institute (JBI) critical appraisal checklist was used to assess the quality of the included studies and ensure their methodological rigour. As shown in Supplementary Table 2.

## Results

### Studies selection

**Fig. 1:**
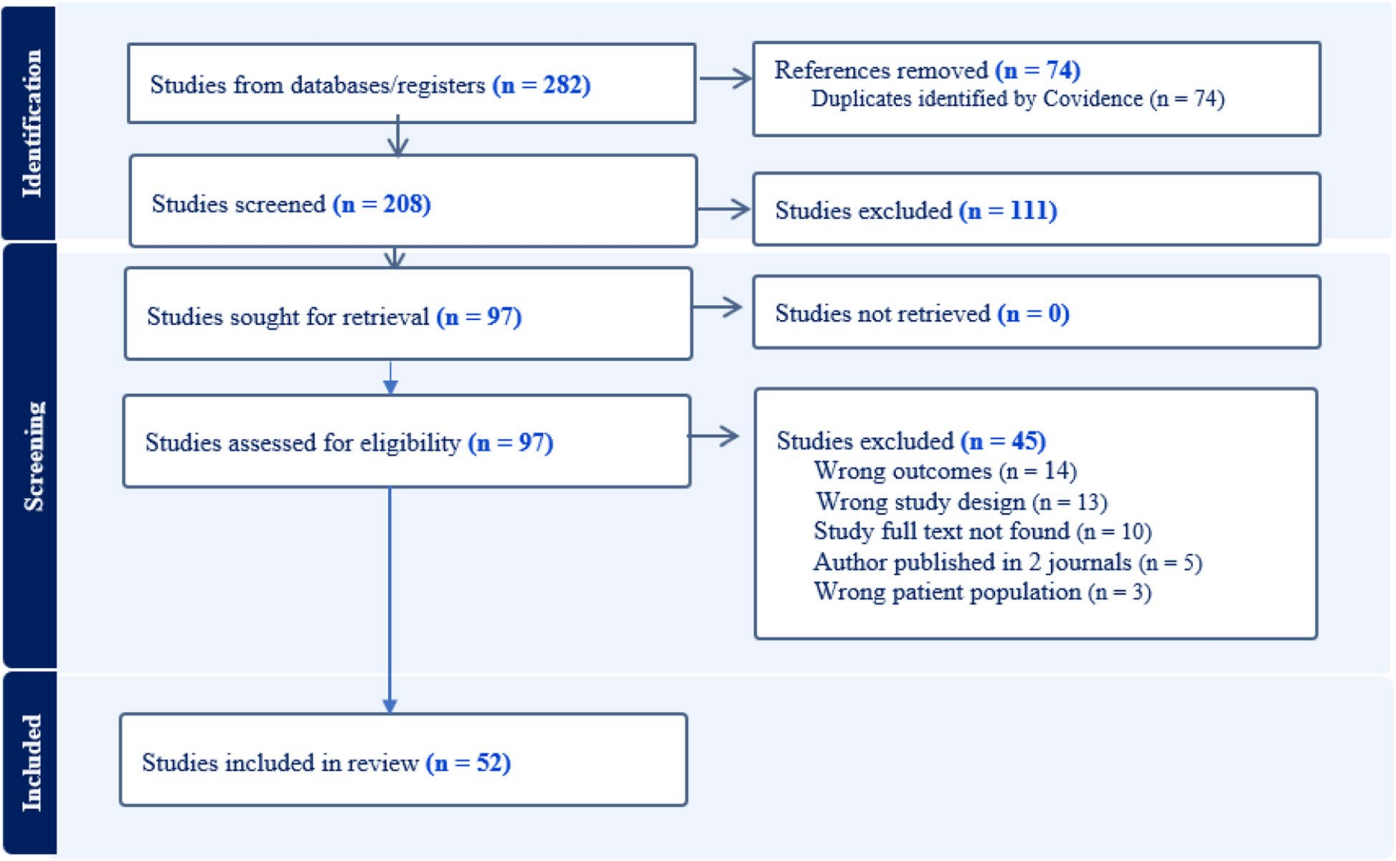
PRISMA flowchart showing the screening process and number of studies included, excluded, duplicates, and irrelevant studies

The systematic database search identified 282 records. After removing 74 duplicate records using Covidence, the remaining 208 articles were employed for title, abstract, and keyword screening. Subsequently, we excluded 111 irrelevant articles. After thoroughly examining the full text of the remaining 97 articles, 52 articles were included in this systematic review. The PRISMA flowchart displays the retrieval process in detail (Fig. 1). All the articles successfully passed the quality assessment, supplementary Table 2.

This review included 52 studies conducted between 2008 and 2024 across all six GCC countries: Saudi Arabia (SA), Oman, United Arab Emirates (UAE), Qatar, Kuwait, and Bahrain. The vast majority (n = 52) employed a cross-sectional design, with one study utilising a quasi-experimental approach, such as surveys or direct interviews. Most were conducted in SA, with study populations ranging from healthcare professionals and university students to the public. Sample sizes ranged from 77 to over 2,500 participants, with age groups primarily between 15 and 65 years. The surveys and interviews targeted medical, dental, applied medical, pharmacy, business, and arts students, as well as secondary school students. The participants in these studies included health care providers, nurses, physicians, parents, attendees of public health centres, and women in communities. Data collection was commonly conducted through online or self-administered questionnaires, while a few studies included interviews or took place in institutional settings such as hospitals, schools, and universities.

### Data extraction

After reviewing the full texts of eligible studies, two independent reviewers extracted the data. Supplementary Table 3 shows the data extracted from the included studies according to standardised form arranged as vaccination uptake and coverage rate, barriers and/or enablers: knowledge, awareness of HPV infection and related cervical cancer, acceptability by parent and medical staff, negative attitudes towards HPV vaccination, vaccine side effects, safety of the vaccines, vaccine availability, vaccine cost, and accessibility. The two reviewers discussed their disagreements, and the third resolved the dispute. The research team discussed and agreed on the final characteristics of the template for data extraction.

#### Knowledge and awareness of HPV and the vaccine

Awareness of CC was relatively high in several studies. Sources of information varied, with the internet, social media, and self-learning (outcome of self-created experience that promotes the decision making of the learners, Knowles, 1975 [22]) being the most frequently reported. In contrast, awareness of HPV infection and the HPVV remained significantly lower. In addition, HPV vaccination programmes vary across the GCC, with some countries successfully implementing them, while others have not.

##### Saudi Arabia

Awareness of CC was relatively high in several studies, reaching ≥ 80% in SA in a few [23]. However, no more than 17.7% of participants displayed prior knowledge of HPVV [24, 25]. It was noted that even among medical students, the understanding of the link between HPV and cervical cancer was poor [25]. However, another study conducted in 2022 showed that 52.3% of medical students believed that the vaccine should be given to both boys and girls, compared to 22.1% of non-medical students [26]. Another study showed that among the 326 surveyed women in primary care clinics, only 21% knew about the association of HPV with CC, and 25% knew about the availability of the vaccine [27]. Another study in 2022 surveyed 1489 women in AL Madinah Province and showed that only 12.6% are aware of HPVV availability [28].

Among the primary care community, 80% of the 200 surveyed physicians recognised that HPVV is essential for public safety, but only 16.5% advised their patients to take it [29]. Among 200 female physicians, after being provided with information about the HPVV, 50% of physicians indicated they would recommend it to their patients [30]. SA, integrated HPV vaccination into the national immunisation schedule in 2019 for females aged 11–26, with optional access for males through Ministry of Health facilities, private providers, and routine outpatient settings [31]. Finally, Al-Shaikh et al. (2017) demonstrated significant increases in knowledge and vaccine acceptance following an educational intervention [32].

##### UAE

In a relatively exhaustive study covering the GCC area, awareness of the HPV vaccine was highest in the UAE (49.6%), and, consistently, awareness of Pap smear testing among females was similarly high (62.4%) [33].

Among 606 females from UAE, their awareness that the HPVV is effective against CC was observed in 26.9%. In the same country, among 390 men, only 16.7% were familiar with HPV [34]; however, among school nurses, 97% were aware of HPV and HPVV [35]. In contrast, healthcare providers were underutilised as information sources, although studies consistently found that participants expressed greater trust in healthcare professionals [36]. A national school-based vaccination programme began in 2008 for girls aged 15–20 years in Abu Dhabi, with coverage exceeding 95% [37].

##### Qatar

Whereas awareness of HPV infection and the HPVV remained significantly lower. Among 397 females in Qatar, 11.8% reported awareness that the HPVV is effective against CC. Qatar approved the vaccine in May 2023 as an optional offering via the Ministry of Public Health [38].

##### Bahrain, Kuwait and Oman

In Bahrain, among the 300 women at primary care clinics, only 3.7% heard about HPVV in 2018 [39]. However, in these three countries, Kuwait, Oman, and Bahrain, the HPV vaccine is not integrated into the national immunisation programmes. Individuals desiring HPV vaccination can obtain it through the private healthcare sector [40–42].

#### HPV Vaccine uptake, coverage and effectiveness

Actual uptake of the HPV vaccine was consistently low across all studies. None of the included studies directly evaluated vaccine coverage or programme effectiveness. In contrast, willingness to receive the vaccine was more promising.

##### Saudi Arabia

HPVV uptake in SA ranged between < 1% and 16% [43, 44]. A study reported 4.6% HPVV uptake [33]. The most shocking was a study conducted in 2022, in Najran City, SA, that surveyed 1085 women and showed that 99% of them did not receive the HPV vaccine [45]. Healthcare provider recommendation, prior hepatitis B vaccination, and higher education levels were associated with greater acceptance.

##### UAE, Qatar, Oman and Kuwait

The UAE showed the highest vaccination rate at 18.9% (50/264) [33]. A high vaccine uptake (> 95%) was reported in Abu Dhabi through its school-based programme [37]. While HPVV uptake reported in Qatar was much lower than in the UAE, it ranged from 5.8% to 7% [33, 38]. Typically, HPVV in Oman remains below 10%, and uptake ranges from 0% to 3.2% [46, 47]. Consistently, both Bahrain and Kuwait HPVV uptake was low 2.9% and from 1.9% to 8.9% respectively [40, 48].

#### Barriers to HPV vaccine uptake

**Fig. 2:**
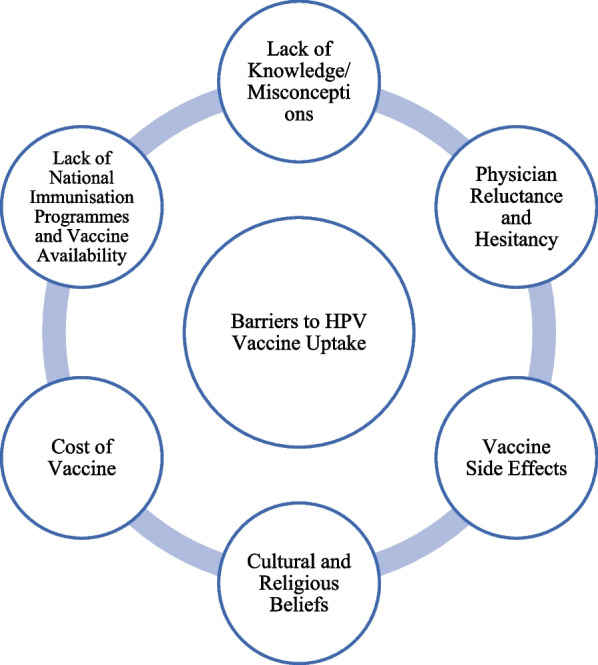
Six themes of barriers to HPV vaccine uptake mentioned in 52 GCC studies

Of the 52 studies, 50 identified barriers that have been grouped into six major themes: lack of knowledge about HPV, concerns over vaccine safety and side effects, cultural and religious objections, and misconceptions that the vaccine promotes sexual activity. Additionally, there is a lack of national immunisation programmes and vaccine availability, cost concerns, and physicians' reluctance to discuss sexual health and disinterest in recommending vaccination (Fig. 2). Other reasons included believing vaccination was unnecessary due to being healthy, lack of doctor recommendation, limited time, and misconceptions that the vaccine was only for females.

##### Saudi Arabia

In SA, the most frequently cited barrier was a lack of knowledge about HPV, its transmission, and the purpose of the vaccine, reported across nearly all studies [24, 49–53]. On the other hand, upon improving this knowledge, the willingness to receive the vaccine increased [24]. Other common barriers included concerns over vaccine safety and side effects [54]. Cultural and religious objections were also identified in the Saudi community [34, 55, 56]. Misconceptions that the vaccine promotes sexual activity are also identified as another significant barrier [57, 58]. Lack of national immunisation programmes and vaccine availability also poses a challenge, as reported by many studies [33, 49, 59]. As well as cost concerns, particularly in settings where the vaccine is not publicly funded [32]. Finally, physicians' passive role in discussing HPV-related disease and transmission vaccine recommendations played a key role in such low awareness and HPVV rate in this area [60].

##### Qatar

In Qatar, physicians' unwillingness to discuss sexual health, disinterest in recommending vaccination, and their hesitancy to be vaccinated were reported in physician-based studies. Another barrier to accepting the HPVV by the community in this country is the lack of awareness about the relationship between HPV and cervical cancer (61.6%), efficacy worries 32.5%, safety concerns 26.9%, triggering the sexual desire and promoting the risky sexual behaviour concerns 26.8%, perceived low-risk 23.3% and cost 24.6%. [61].

##### UAE & Oman

In the UAE, common barriers included concerns over vaccine safety and side effects [34]. As well as cost concerns, particularly in settings where the vaccine is not publicly funded [37]. While in Oman, misconceptions that the vaccine promotes sexual activity were reported [46].

#### Enablers for HPV vaccine uptake

**Fig. 3:**
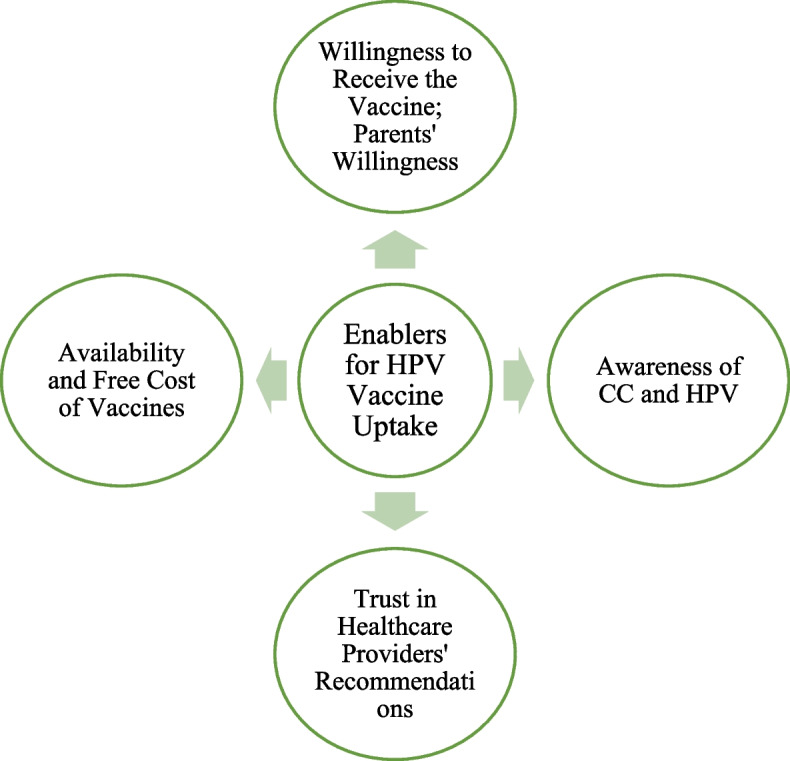
Most frequently cited enablers for HPV vaccine uptake in 39 GCC studies

Thirty-nine studies described enablers of HPV vaccine uptake. The most frequently cited enabler was participants’ willingness to receive the vaccine. While facilitating awareness of HPV and CC and their prevention is education, it came as the second most frequent enabler. This includes personal knowledge of the conditions, finding information on HPV prevention online or through educational programmes, integrating awareness campaigns into the HPV vaccination programme, and promoting initiatives to help overcome barriers to vaccination. Healthcare providers also played a key role, with participants expressing a preference for vaccine recommendations from doctors (Fig. 3).

##### Saudi Arabia

The most frequently cited enablers in SA were participants’ willingness to receive the vaccine [62, 63], and parental willingness to vaccinate daughters was also widely reported [51, 64]. Al-Darwish et al. in 2014 reported that the sources of information for medical students were self-learning, curriculum, faculty, hospital and internet [25]. Furthermore, Al-Shaikh et al. in 2017 compared the knowledge among the surveyed students before and after the educational programme intervention, and the results were positive and encouraging [32] Physician support for integrating the HPVV into immunisation schedules was also substantial [43, 44, 52, 64, 65]. The physician sample, for example, in Almazrou et al.'s study, was found to be positive, encouraging women to take up the vaccine for healthcare women seekers and even their daughters (the physician's daughters) [64].

##### Oman

The most frequently cited enabler in Oman was participants’ willingness to receive the vaccine, while the sources of information for participants were friends, the internet, and social media [41]. About 60% of respondents in Al Alawi et al. (2023) and Al-Saadi et al. (2021) expressed readiness to be vaccinated, especially if the vaccine were offered free of charge [41, 66].

##### Qatar & UAE

Participants’ readiness to receive the vaccine and free vaccine delivery were introduced as enablers in Qatar and the UAE, which were decisive factors in the latter's vaccine acceptance. Finally, the recommendation by the Ministry of Health is another critical enabler in the UAE [37, 61, 67, 68].

#### Cost-effectiveness of HPV vaccination

##### Saudi Arabia & Oman

Only two studies assessed cost-effectiveness, one in Oman and one in Saudi Arabia [69, 70]. Both concluded that HPV vaccination was not cost-effective due to low cervical cancer incidence and high vaccine costs. However, these conclusions were limited by the absence of national screening programmes and insufficient HPV prevalence data. Further research is needed to inform cost-effectiveness evaluations in the GCC context

#### Recommendations by included studies

##### Saudi Arabia

Most recommended actions were raising awareness of HPV infection [65, 71]. The importance of the vaccine is highlighted through health education campaigns, the integration of HPV topics into school and university curricula, and culturally sensitive outreach [24, 32].

##### UAE, Oman, Bahrain, & Kuwait

HPV infection awareness and the integration of the HPV vaccine into national immunisation schedules are advocated, particularly in Oman [36, 42, 46, 48, 72].

## Discussion

This systematic review evaluated the literature on the HPV vaccination knowledge, attitudes, barriers, enablers, and policy recommendations in GCC countries. The findings indicate significant regional disparities in awareness, uptake, and implementation strategies, underscoring the need for context-specific interventions to improve HPV-related health outcomes.

Knowledge of HPV infection and its vaccine remained markedly limited, although awareness of CC was relatively high in many studies. This knowledge gap was consistent across diverse populations, including the public, students in health disciplines, and healthcare providers. Studies in Saudi Arabia and Oman [24, 73] consistently reported that fewer than 30% of participants had heard of the HPV vaccine, with even lower proportions aware of its preventive role. This disconnect is concerning, given the established causal link between persistent HPV infection and cervical cancer, as well as other HPV-associated malignancies [2].

Despite some willingness to receive the vaccine, often exceeding 60% in multiple studies, the actual uptake was alarmingly low. In most studies, HPV vaccine coverage was reported at less than 10%, with some studies, such as Abu Sanad et al. (2024) and Rezqalla et al. (2021), reporting uptake rates as low as 1.9–2.0% [23, 40]. This gap between intention and action highlights systemic and individual-level barriers, including limited access, insufficient public health infrastructure, and weak policy enforcement.

In contrast, the UAE’s school-based HPV programme in Abu Dhabi has demonstrated greater success, with uptake exceeding 95% following the introduction of free, school-based vaccination [37]. This suggests institutional delivery mechanisms and financial support are critical levers for improving coverage.

This study showed different rates of HPV vaccine uptake among GCC countries. Abu Dhabi showed the highest uptake rates, rising from 53% in 2011 to > 95% in the 1st quarter of 2013. Ortashi et al. [74] explained that the rise in HPV vaccination uptake can be attributed to broad public and parent awareness initiatives and the training of healthcare providers [74]. In addition, this is attributable to the school-based approach. This is in line with Donken et al. in 2021, who reported a significant annual drop in rates of Cervical Intraepithelial Neoplasia (CIN) CIN2 and CIN3 that occurred following the establishment of the school-based HPV vaccination initiative among females aged 16 to 23, who were the target cohorts for the vaccination programme [75]. Moreover, this is supported by a study by Lim et al. (2014), which found a high completion rate for the three-dose HPV vaccine series through a publicly funded school programme [76]. Consistently, Patel et al. 2016 indicated that an almost 100% coverage rate for the first and second doses of the bivalent HPV vaccine and 93% for the third dose was achieved in Salta (a province in Argentina) through a school-based programme [77]. Schools are places with large numbers of students and their families and are always among the most trusted institutions in the community [78]. In addition, teachers and management are very influential factors in guiding students at these ages to their well-being.

In SA, vaccine uptake varied from under 1% to 16%, likely underestimating actual rates due to the focus of studies on both intended and unintended cohorts, such as older adolescents and males, who were not expected to be vaccinated. Health centres have proven effective for delivering the HPV vaccine, as shown by Patel et al. in 2016, who reported high coverage rates in Buenos Aires when administered in health centres instead of schools [77]. In fact, both school-based strategies (such as on-site vaccination, partnerships with clinics, and student engagement) and communication by healthcare centre professionals are among the effective strategies to increase HPV vaccine uptake. Barriers such as a lack of awareness and cultural sensitivity may contribute to the low uptake. Specific concerns were raised by the parents, who were more concerned about the cultural and religious aspects, and vaccine uptake could encourage sexual activity.

The findings reveal several barriers that hinder vaccine uptake. The most prominent was a lack of knowledge about HPV and the vaccine, cited in nearly all studies. Misconceptions, such as the belief that vaccination promotes promiscuity, alongside concerns about vaccine safety and efficacy, were also common. Cultural sensitivities, religious reservations, cost, and the absence of national immunisation programmes were recurrent themes across studies conducted in SA, Oman, Bahrain, and Qatar [34, 46, 61]. This observation aligns with global research conducted by Zheng et al. in 2021 [79], which showed adolescents' reluctance to initiate HPV vaccination programmes was influenced by insufficient understanding of HPV, cervical cancer, and HPV vaccines [79]. Kutz et al. in 2023 also showed that insufficient awareness about vaccination services and a restricted understanding of HPV and its development were recognised as obstacles [80]. This matched with a study conducted in the East Mediterranean Regional Office (EMRO) region by Hakimi et al. in 2023, stating that insufficient awareness poses a main barrier to adopting HPV vaccination [19].

Healthcare providers, although viewed as trusted sources, were often underutilised due to their own knowledge gaps or discomfort discussing sexually transmitted infections. Physician-specific studies (e.g. [60, 61]) highlighted reluctance to recommend the vaccine due to perceived moral implications, limited clinical training, or sociocultural discomfort, factors that must be addressed through continued medical education.

Concern about vaccine safety, efficacy, and side effects was the second most reported barrier. This concern is mainly due to misinformation regarding vaccines, as mentioned by Kutz [80]. However, since the first FDA approval of the HPV vaccine in 2006, more than 500 million doses of HPV vaccines have been used with no serious safety matter announced apart from rare reports of anaphylaxis. The vaccine's safety profile continues to be reassuring in universal use from all sources of information [2]. This provides an opportunity to spread correct information on social networks and initiate an educational campaign for the public. Similarly, Zheng et al. in 2021 [79] stated that the most prevalent reason for declining HPV vaccinations among individuals aged 9 to 26 years was apprehension about adverse effects [79].

Another barrier is the cost of vaccination; free vaccine provision positively affects the initiation of HPV vaccination. In Kuwait, the HPVV uptake was just up to 8.9%, which could be attributed to the vaccine cost because it is only available through the private sector. This is supported by Holman et al. in 2014, who indicated that vaccine cost is a barrier to HPVV delivery to the target population [81]. Cost concerns results agree with those of Elbarazi et al. in 2016, who documented that higher vaccine uptake rates were observed in public government schools compared to private schools in Abu Dhabi, as the vaccine is free for Emirati citizens [82]. This is comparable to the finding of Sundaram et al. in 2021, who conducted a study among female expatriate students at a private university in the UAE, where only 5% of those surveyed had received the vaccination, which may be attributed to the vaccine cost [72]. Broadly, in places where the vaccine is free of charge, higher HPV vaccination coverage rates are noticed [79].

Several studies identified promising enablers for HPV vaccination, including willingness to be vaccinated, increased health literacy, and trust in healthcare providers. Positive attitudes were particularly noted among participants with prior exposure to related health information, such as those who had received the hepatitis B vaccine or studied health sciences [25, 44]. Educational interventions were shown to be effective in raising awareness and correcting misconceptions, as demonstrated by Al-Shaikh et al. [32]. Akkour et al. in 2021 demonstrated that 54% of the surveyed women were willing to be vaccinated after receiving the information [24].

The third most common enabler was healthcare providers’ awareness. This was explained by Ortashi et al. in 2013 [37], who showed that although there was limited awareness of HPV and the vaccine, there was widespread vaccine acceptance [37]. This could be attributed to the public's strong trust in recommendations from the government and healthcare providers. This agrees with Zheng et al. in 2021 [79], who revealed that adolescents rely more on the recommendations given by healthcare providers regarding the vaccine than from other sources [79].

Cost was a frequently cited barrier, but studies also found that free vaccine provision significantly improved acceptability. In the UAE and Qatar, support for government-subsidised vaccination was high, and a majority of participants indicated willingness to vaccinate themselves or their children if the vaccine were offered free of charge [36, 61].

Most studies called for multifaceted public health strategies. Key recommendations included integrating the HPV vaccine into national immunisation schedules, especially in countries where the vaccine is only available in the private sector and launching targeted awareness campaigns. Culturally sensitive health promotion, delivered through schools, universities, and healthcare providers, was recommended to overcome stigma and improve uptake.

Institutional reforms were also proposed. These included reviewing medical and nursing curricula to enhance HPV training, improving provider communication skills regarding sexual health, and establishing national registries to monitor vaccine coverage and outcomes. Importantly, several studies underscored the need for further research to understand local epidemiology and inform cost-effectiveness analyses, which remain limited in the region.

While this review provides valuable insights, it also highlights several evidence gaps. First, few studies assessed the effectiveness of implemented vaccination programmes or interventions beyond self-reported knowledge and attitudes. Second, limited qualitative data explores deeper cultural, familial, and gender-specific influences on vaccine behaviour. Lastly, male populations remain underrepresented in HPV research, despite increasing evidence of HPV-related morbidity in men. Due to the heterogeneity of the studies included, comparing the results related to the vaccine uptake was not optimal. One of the objectives was to assess the effectiveness of the HPV vaccination programme; sadly, we did not find data comparing the HPV prevalence before and after the vaccine implementation. We also aimed to identify the characteristics of those who received the vaccine and where they received it, but no study has addressed this aspect. As GCC countries continue to invest in preventive health strategies aligned with national visions for health and development, addressing the structural, social, and informational determinants of HPV vaccine uptake is essential. Future research should prioritise intervention studies, cost-effectiveness analyses, and longitudinal evaluations to better guide policy and practice. Strengthening HPV prevention efforts will be vital to reducing the burden of cervical cancer and other HPV-related diseases across the region.

### Policy recommendations

Given the similarity in culture, economy, climate, and health system, this study provides more substantial evidence that shared enablers and challenges arose across all six communities, which encourage policymakers to adopt and tackle them. The findings of this review underscore the urgent need for policy change. It is recommended that policymakers across GCC countries give precedence to incorporating the HPV vaccine within national immunisation programmes. This should be reinforced by strengthening school-based health initiatives, which have demonstrated effectiveness as a delivery platform. In parallel, sustained investment is required to advance the training of healthcare providers, encourage ongoing community participation, and implement comprehensive public health campaigns aimed at reducing knowledge deficits and addressing cultural sensitivities.

## Conclusion

Our review demonstrates the notable difference in the HPV vaccine uptake rate among the various approaches in GCC countries, with the school-health strategy showing the highest success rate in Abu Dhabi. Although there is moderate awareness of cervical cancer, knowledge of HPV infection and its vaccine remains insufficient across both the public and healthcare professional populations. Actual vaccine uptake is low, despite notable willingness to vaccinate, particularly when cost and access barriers are removed.

This review highlights a range of multilevel barriers, including lack of knowledge, safety concerns, cultural sensitivities, and absence of national immunisation programmes, as well as key enablers such as trust in healthcare providers, targeted education, and free vaccine provision. This research encourages decision-makers in GCC countries to support the integration of the HPV vaccine into the national vaccination programme, with further emphasis on school health programmes, enhanced health professional training, sustained community engagement, and vaccine campaigns.

## Supplementary Information

Supplementary Material 1.

Supplementary Material 2.

Supplementary Material 3.

## Data Availability

All data supporting the findings of this study are available within the paper and its Supplementary Information.

## Abbreviations

PHC: Primary health care
HPV: Human papillomavirus
HPVV: Human papillomavirus vaccine
CC: Cervical cancer
GCC: Gulf Cooperation Council
DNA: Deoxyribonucleic Acid
HR: High risk
LR: Low risk
CIN: Cancerous intra-epithelial neoplasia
STI: Sexually transmitted infection
HIV: Human immunodeficiency virus
NAAT: Nucleic acids amplification test
Pap: Papanicolaou
VLP: Virus-like particle
WHO: World Health Organization
JBI: Joanna Briggs Institute
