# Supplementary material for: Knowledge and awareness of HPV vaccination uptake and recommendations in gulf cooperation council countries 2009–2025: a systematic review
Source: Arch Public Health. 2026 Mar 13;84:81. doi: 10.1186/s13690-026-01875-6 (PMC13097827; doi:10.1186/s13690-026-01875-6)
Supplement: Supplementary file 1 — Supplementary Material 1. [file 13690_2026_1875_MOESM1_ESM.docx]

Supplementary Table 1: Search Strings Summary

| **Database** | **Platform** | **Search Terms (Keywords & Controlled Vocabulary)** | **Limits Applied** | **Date Searched** |
| --- | --- | --- | --- | --- |
| Ovid MEDLINE | Ovid | “human papillomavirus” OR “HPV” OR “HPV vaccin*” OR “human papillomavirus vaccin*” AND (“awareness” OR “knowledge” OR “attitudes” OR “uptake” OR “barrier*”) AND (Saudi Arabia OR GCC country names) | English/Arabic; Human; GCC countries | 11 March 2025 |
| Ovid Embase | Ovid | As above, with adapted Emtree terms (e.g. 'human papillomavirus'/exp, 'vaccination'/exp) | As above | 11 March 2025 |
| APA PsycINFO | Ovid | “HPV” OR “human papillomavirus” AND “attitudes” OR “acceptance” OR “awareness” OR “beliefs” AND GCC country names | As above | 11 March 2025 |
| Global Health | Ovid | “HPV vaccin*” OR “cervical cancer prevention” AND “barrier*” OR “enabler*” AND GCC countries | As above | 11 March 2025 |
| Maternity and Infant Care Database | Ovid | “HPV” OR “HPV vaccine” AND “vaccination uptake” OR “parental acceptance” AND (Saudi Arabia OR UAE OR Oman OR Qatar OR Kuwait OR Bahrain) | As above | 11 March 2025 |
| Health Management Information Consortium (HMIC) | Ovid | “HPV immunisation” OR “vaccine coverage” OR “health policy” OR “programme implementation” AND GCC countries | As above | 11 March 2025 |
| PubMed | NCBI | (“HPV” OR “human papillomavirus”) AND (“vaccination” OR “vaccine uptake” OR “knowledge”) AND (“barriers” OR “facilitators”) AND (Saudi Arabia OR UAE OR other GCC countries) | English; | 11 March 2025 |
| Cochrane Central Register of Controlled Trials (CENTRAL) | Cochrane | “HPV vaccine” OR “cervical cancer prevention” AND “awareness” OR “intervention” AND GCC countries | Trials; Human studies | 11 March 2025 |
| WHO Global Index Medicus | WHO | “HPV” OR “cervical cancer” AND “vaccine” OR “programme” AND “Middle East” OR specific GCC country names | English; | 11 March 2025 |
| Web of Science Core Collection | Clarivate | TS=(HPV OR “human papillomavirus”) AND TS=(vaccine OR vaccination OR awareness OR uptake OR barrier*) AND TS=(Saudi Arabia OR Oman OR UAE OR Kuwait OR Qatar OR Bahrain) | English; Articles only; | 11 March 2025 |
