# Supplementary material for: Knowledge and awareness of HPV vaccination uptake and recommendations in gulf cooperation council countries 2009–2025: a systematic review
Source: Arch Public Health. 2026 Mar 13;84:81. doi: 10.1186/s13690-026-01875-6 (PMC13097827; doi:10.1186/s13690-026-01875-6)
Supplement: Supplementary file 2 — Supplementary Material 2. [file 13690_2026_1875_MOESM2_ESM.docx]

Supplementary Table 2: JBI Quality Assessment of 52 Included Studies.

|  | **Study ID / Citation** | **1) Were the criteria for inclusion in the sample clearly defined?** | **2) Were the study subjects and the setting described in detail?** | **3) Was the exposure measured in a valid and reliable way?** | **4) Were objective, standard criteria used for measurement of the condition?** | **5) Were confounding factors identified?** | **6) Were strategies to deal with confounding factors stated?** | **7) Were the outcomes measured in a valid & reliable way?** | **8) Was appropriate statistical analysis used?** | **Overall appraisal** |
| --- | --- | --- | --- | --- | --- | --- | --- | --- | --- | --- |
| 1 | (Akkour et al., 2021) | Y | Y | Y | Y | Y | N/A | Y | Y | include |
| 2 | (Al Alawi et al., 2023) | Y | Y | Y | Y | Y | Y | Y | Y | include |
| 3 | (Al-Darwish et al.,2014) | Y | Y | Y | Y | Y | Y | Y | Y | include |
| 4 | (Aldawood et al., 2023) | Y | Y | Y | Y | Y | N/A | Y | Y | include |
| 5 | (Aldohaian et al., 2019) | Y | Y | Y | Y | Y | Y | Y | Y | include |
| 6 | (Ali et al., 2022) | Y | Y | Y | Y | Y | Y | Y | Y | include |
| 7 | (Al Kalbani et al., 2022) | Y | Y | Y | Y | Y | N/A | Y | Y | include |
| 8 | (Almazrou et al., 2020) | Y | Y | Y | Y | Y | N | Y | Y | include |
| 9 | (Almehmadi et al., 2019) | Y | Y | Y | Y | Y | N/A | Y | Y | include |
| 10 | (Almughais et al., 2018) | Y | Y | Y | Y | Y | N/A | Y | Y | include |
| 11 | (Alnafisah et al., 2019) | Y | Y | Y | Y | Y | N/A | Y | Y | include |
| 12 | (Al-Nuaimi et al., 2011) | Y | Y | Y | Y | Y | Y | Y | Y | include |
| 13 | (AlObaid et al., 2014) | Y | Y | Y | Y | Y | N/A | Y | Y | include |
| 14 | (Al Raisi et al., 2022) | Y | Y | Y | Y | Y | N/A | Y | Y | include |
| 15 | (Alrajeh and Alshammari, 2020) | Y | Y | Y | Y | Y | N/A | Y | Y | include |
| 16 | (Al-Saadi et al., 2021) | Y | Y | Y | Y | Y | Y | Y | Y | include |
| 17 | (Alsanafi et al., 2023) | Y | Y | Y | Y | Y | N/A | Y | Y | include |
| 18 | (Al Sekri et al., 2021) | Y | Y | Y | Y | Y | N | Y | Y | include |
| 19 | (Al-Shaikh et al., 2014) | Y | Y | Y | Y | Y | Y | Y | Y | include |
| 20 | (Al-Shaikh et al., 2017) | Y | Y | Y | Y | Y | Y | Y | Y | include |
| 21 | (Alshammari and Khan, 2022) | Y | Y | Y | Y | Y | Y | Y | Y | include |
| 22 | (Al Shdefat et al., 2022a) | Y | Y | Y | Y | Y | N | Y | Y | include |
| 23 | (Al Shedfat et al., 2022b) | Y | Y | Y | Y | Y | N | Y | Y | include |
| 24 | (Alsous et al., 2021) | Y | Y | Y | Y | Y | N/A | Y | Y | include |
| 25 | (Sait, 2011) | Y | Y | Y | Y | Y | N | Y | Y | include |
| 26 | (Anfinan, 2019) | Y | Y | Y | Y | Y | Y | Y | Y | include |
| 27 | (Azer et al., 2022) | Y | Y | Y | Y | Y | Y | Y | Y | include |
| 28 | (Darraj et al., 2022) | Y | Y | Y | Y | Y | Y | Y | Y | include |
| 29 | (Dhaher, 2019) | Y | Y | Y | Y | Y | Y | Y | Y | include |
| 30 | (Elgzar et al., 2022) |  | Y | Y | Y | Y | N/A | Y | Y | include |
| 31 | (Farsi et al., 2020) | Y | Y | Y | Y | Y | N | Y | Y | include |
| 32 | (Farsi et al., 2021) | Y | Y | Y | Y | Y | Y | Y | Y | include |
| 33 | (Gari et al., 2012) | Y | Y | Y | Y | Y | N/A | Y | Y | include |
| 34 | (Husain et al., 2019) | Y | Y | Y | Y | Y | Y | Y | Y | include |
| 35 | (Ibrahim et al., 2022) | Y | Y | Y | Y | Y | N/A | Y | Y | include |
| 36 | (Jassim et al., 2018) | Y | Y | Y | Y | Y | N/A | Y | Y | include |
| 37 | (Jradi and Bawazir, 2019) |  | Y | Y | Y | Y | N | Y | Y | include |
| 38 | (Hendaus et al., 2021) |  | Y | Y | Y | Y | N/A | Y | Y | include |
| 39 | (Ortashi et al., 2014) | Y | Y | Y | Y | Y | N/A | Y | Y | include |
| 40 | (Ortashi et al., 2013) | Y | Y | Y | Y | Y | N | Y | Y | include |
| 41 | (Ortashi et al., 2012) | Y | Y | Y | Y | Y | N | Y | Y | include |
| 42 | (Rezqalla et al., 2021) | Y | Y | Y | Y | Y | Y | Y | Y | include |
| 43 | (Sait, 2009) | Y | Y | Y | Y | Y | N | Y | Y | include |
| 44 | (Saqer et al., 2017) | Y | Y | Y | Y | Y | N/A | Y | Y | include |
| 45 | (Sundaram et al., 2021) | Y | Y | Y | Y | Y | N | Y | Y | include |
| 46 | (Tehsin et al., 2021) | Y | Y | Y | Y | Y | N/A | Y | Y | include |
| 47 | (Zahid et al., 2022) | Y | Y | Y | Y | Y | N | Y | Y | include |
| 48 | (Mahmoud et al., 2024) | Y | Y | Y | Y | Y | Y | Y | Y | include |
| 49 | (Cheema et al., 2024) | Y | Y | Y | Y | Y | Y | Y | Y | include |
| 50 | (Alosaimi et al., 2024) | Y | Y | Y | Y | Y | Y | Y | Y | include |
| 51 | (Albayat et al., 2024) | Y | Y | Y | Y | Y | Y | Y | Y | include |
| 52 | (Abu Sanad et al., 2024) | Y | Y | Y | Y | Y | Y | Y | Y | include |
