# Supplementary material for: Knowledge and awareness of HPV vaccination uptake and recommendations in gulf cooperation council countries 2009–2025: a systematic review
Source: Arch Public Health. 2026 Mar 13;84:81. doi: 10.1186/s13690-026-01875-6 (PMC13097827; doi:10.1186/s13690-026-01875-6)
Supplement: Supplementary file 3 — Supplementary Material 3. [file 13690_2026_1875_MOESM3_ESM.docx]

Supplementary Table 3: Extraction Table for 52 GCC Studies.

|  |  |  |  |  |  |  |  |  |  |
| --- | --- | --- | --- | --- | --- | --- | --- | --- | --- |
|  | **References (author and year)** | **Country** | ***M*ain *A*im** | **Type of study, Duration, and Setting** | **Sample *S*ize and *A*ge** | **Outcome *M*easure** | **Barriers** | **Enablers** | **Opportunities and *R*ecommendations** |
| 1 | (Akkour et al., 2021) | Saudi Arabia | Evaluate the public awareness of cervical cancer, (CC), risk factors, HPV infection, and HPV vaccines in different regions of Saudi Arabia | A cross-sectional self-administered study was distributed through different social media platforms.    -Duration 1 month | 564 Saudi women (18-64 years old). | 84.0% showed awareness of CC.  -The internet was the main information source.  78.9% were unaware of the sexual transmission of HPV.  81.9% were unaware of HPV's link to CC.  17.7% had prior knowledge of the HPV vaccine.  2.0% received the vaccine.  -After receiving information, 54.1% expressed willingness to get vaccinated. | -Lack of knowledge about HPV infection & HPVV. | Client’s awareness about CC, HPV & HPVV.  -Willingness to receive the HPVV. | -Initiation of HPV vaccination program.  -Raising awareness regarding HPV & related disease & HPVV. |
| 2 | (Al Alawi et al., 2023) | Oman | Assess knowledge, attitudes, and acceptance toward HPV vaccination in men and women in Oman. | A cross-sectional quantitative study through a validated self-administered questionnaire online.  -Duration: three months (May–July 2021) | A total of 1403 participants, including 952 parents and 369 healthcare providers, completed the survey (aged 18 years and above). | -Limited awareness about HPV infection and vaccines.  25% were aware of HPV.  -The main source of information was the internet.  -The majority had worries about vaccine safety, side effects, and the vaccine's effectiveness.  66% of the participants agreed to receive the HPVV, expressing support for vaccinating both boys and girls. | -Lack of knowledge about HPV infection & HPVV.  -Vaccine safety, efficacy & side effects. | Willingness to receive the HPVV.  -Client’s awareness about CC, HPV & HPVV  -Health care providers’ awareness about CC, HPV & HPVV. | -. Initiation of HPV vaccination program.  -Raising awareness regarding HPV & related disease & HPVV. |
| 3 | (Al-Darwish et al.,2014) | Saudi Arabia | Assess the knowledge regarding symptoms, risk factors and prevention of cervical carcinoma among medical students in the Kingdom of Saudi Arabia. | -Cross-sectional study by self-administered questionnaire.  -Duration: 6 months, (December 2012 to May 2013). | -188 medical students in their second, third, fourth, and fifth year of study at the College of Medicine, King Faisal University, Al-Ahsa, KSA. | 55% of males and 46.8% of females could not link HPV infection to CC.  67% were unaware of the availability of HPVV.  -The main source of knowledge was self-learning, followed by the medical school curriculum. | - Lack of knowledge about HPV infection & HPVV. | -Client’s awareness about CC, HPV & HPVV. | -Raising awareness regarding HPV & related disease & HPVV. |
| 4 | (Aldawood et al., 2023) | Saudi Arabia | -Assessing HPV awareness and knowledge among health college students at King Saud University.  -Comparing these outcomes across sociodemographic characteristics. | -A cross-sectional study by self-administered questionnaire.    -Duration: 2 months (November to December 2022) | -403 participants (aged 18 yrs to ≥ 27 yrs)  from the five health colleges: College of Medicine, Dentistry, Pharmacy, Applied Medical Sciences, and Nursing at King Saud University | 60% were familiar with HPV.  -Medical students showed higher awareness levels (91.8%) than students from other colleges.  Students who received the hepatitis B vaccine were more aware of HPV than students who hadn't been vaccinated. | - Lack of knowledge about HPV infection & HPVV. | -Client’s awareness about CC, HPV & HPVV.  - Health care providers’ awareness about CC, HPV & HPVV. | -. Initiation of HPV vaccination program.  -Raising awareness regarding HPV & related disease & HPVV. |
| 5 | (Aldohaian et al., 2019) | Saudi Arabia | -Assess women’s beliefs about CC and the Pap smear test.  -Evaluate the relationship between CC and the social and demographic characteristics. | -A descriptive cross-sectional study by self-administered questionnaire.  -Duration: 1 and & a half months, from January 15, 2018, to February 30, 2018.  -Setting: some of Saudi Arabia’s major hospitals offer the HPV vaccine based on a doctor’s prescription – women's gynaecology outpatient clinics in Riyadh’s four main hospitals (King Khaled University, Alyamamah, King Saud Hospital, and the King Fahad Medical City) and primary care centres in Riyadh. | 450 participants.  -aged 18 years or older. | 1% received the vaccine.  -The main reason for vaccine refusal was a (91.1%) lack of awareness, (6.4%) related to other factors like infection fears, parental or spousal objections, or vaccine cost.  57.3% were unaware of the suitable age for vaccination.  -The average age for receiving the HPV vaccine among those who had been vaccinated was 29.9 ± 8.6 years. | -Absence of a national HPV vaccination program.  -Lack of knowledge about HPV infection & HPVV.  -Vaccine safety, efficacy & side effects.  -Vaccine cost. | -------- | -. Initiation of the HPV vaccination program.  -Raising awareness regarding HPV & related disease & HPVV. |
| 6 | (Ali et al., 2022) | Saudi Arabia | Assess the knowledge, attitude, and practice toward HPV amongst pharmacy students in Saudi Arabia. | -A prospective cross-sectional study through online survery.  -Duration: two months, from 1 January to 28 February 2020. | -125 pharmacy students at a Mohammed Al-Mana College for Medical Sciences (MACHS).  -student of 2^nd^ year to the 5th year.  -Aged 18 yrs to ≥25 yrs. | 92.8% were aware about CC.  65.0% lacked awareness regarding HPVV.  - Friends and family were the source of information for 19.3%.  43.2% worried that the vaccine may cause complicated sexual behaviour.  -Participants aged 18-25 years and those in the fourth and fifth academic years showed notable knowledge about HPV.  32.8% agreed to be vaccinated due to their trust in their doctors.  32% will take HPVV if it were freely available | -Lack of knowledge about HPV infection & HPVV.  - Misconception that receiving HPVV may encourage risky sexual behaviour. | -Client’s awareness about CC, HPV & HPVV.  -Willingness to receive the HPVV.  -Health care providers’ awareness about CC, HPV & HPVV.  -Vaccine provision free of charge. | - Raising awareness regarding HPV & related disease & HPVV |
| 7 | (Al Kalbani et al., 2022) | Oman | -Assess knowledge, attitudes, and screening practices related to CC & (Pap) smear testing among Omani women who were visitors to a family medicine and public health (FMPH) clinic.  -Correlate the above with the subjects’ sociodemographic characteristics | -A cross-sectional study through an online, structured, self-administered, Arabic-language questionnaire.  -Duration: 14 months, from February 2020 to April 2021. | -Size: 285 Omani women, -Aged ≥ 18 years old. | 89.8% are aware of CC.  71.6% are unaware of HPV or its vaccine.  43.9% support the administration of HPVV to middle school-aged girls.  - The Internet was the main source of information (89.8%), followed by social media (47.4%).  35.4% recognised HPV infection as a risk factor for CC. | -Lack of knowledge about HPV infection & HPVV.  -Misconception that receiving HPVV may encourage risky sexual behaviour | -Client’s awareness about CC, HPV & HPVV.  -Willingness to receive the HPVV | - Initiation of HPV vaccination program.  -Raising awareness regarding HPV & related disease & HPVV. |
| 8 | (Almazrou et al., 2020) | Saudi Arabia | assess the knowledge, attitudes, and practices of physicians. | -A cross-sectional study through online questionnaire.  -Duration: 5 months, between September 2017 and January 2018. | Size: 173 physicians (paediatricians and family medicine physicians working in King Abdul-Aziz Medical City Central Region outpatient clinics at the time of the study (aged 30 to ≥40) | -Most physicians showed a strong understanding of CC & HPV.  3.47% received HPVV.  20% believed that HPVV might encourage early sexual activity activities.  80% believed it is important for women to receive the HPVV, and 82% expressed their willingness to allow their daughters to receive the HPVV.  -Low parental awareness about HPV created a barrier for vaccination, also impacting HPV and HPV vaccine understanding | -Vaccine safety, efficacy & side effects.  - Lack of knowledge about HPV infection & HPVV.  - Misconception that receiving HPVV may encourage risky sexual behaviour. | -Client’s awareness about CC, HPV & HPVV  -Health care providers’ awareness about CC, HPV & HPVV. | -Raising awareness regarding HPV & related disease & HPVV. |
| 9 | (Almehmadi et al., 2019) | Saudi Arabia | determine HPV infection, cervical cancer, and vaccine awareness among the Saudi population. | -cross-sectional study through web-based self-administrated questionnaire.  -Duration: 5 months, between August 2018 and January 2019. | Sample size: 1033, Aged 15- >46 yrs | <16% were aware about HPV.  66% of males and 53.5% of females recognize HPV as a STI.  -A significant number of both genders believed that HPV might lead to complications during pregnancy.  -Both males and females suggested that the HPV vaccine should be accessible to women regardless of marital status. | -Lack of knowledge about HPV infection & HPVV. | -Client’s awareness about CC, HPV & HPVV | -Raising awareness regarding HPV & related disease & HPVV. |
| 10 | (Almughais et al., 2018) | Saudi Arabia | -Evaluate primary health care (PHC) physicians’ awareness of HPV infections and vaccination in Saudi Arabia. | -A cross-sectional study through self-administered survey.  -Duration: 4 months, between May and August 2017.  -Setting: Eleven PHC clinics are in Saudi Arabia. | -Size: 200 PHC physicians, median age 38.8±4.3 years | 80% recognised that HPVV is essential for public safety.  16.5% advised their patients to receive the HPVV.  -The Percentage Mean Score (PMS) for understanding HPV infections was 61.8±10.4, while the PMS for HPVV knowledge was 91.3±11.4.  -Physicians with higher knowledge were more likely to recommend HPVV to their patients. | -Lack of knowledge about HPV infection & HPVV. | - Health care providers’ awareness about CC, HPV & HPVV. | -Raising awareness regarding HPV & related disease & HPVV. |
| 11 | (Alnafisah et al., 2019) | Saudi  Arabia | -Assess the knowledge and attitudes toward screening.  -Determine the status of awareness among women. | -A descriptive cross-sectional study through online self-administered questionnaire  -Duration: two months.  -Setting: Saudi women residing in the Qassim region (central Saudi Arabia) | -Size: 2,220  -aged between 15 and 65 years old. | 70% were aware of CC.  95.6% agreed that CC is treatable.  92.8% were unaware of HPV.  68.6% believed in the efficacy of HPVV.  12.9% recognised that prolonged contraceptive use is a risk factor.  -The internet was the main source of information for 36.8% of participants.  70.8% demonstrated a willingness to provide their daughters with the HPVV. | -Lack of knowledge about HPV infection & HPVV. | -willingness to receive HPVV. | -Raising awareness regarding HPV & related disease & HPVV. |
| 12 | (Al-Nuaimi et al., 2011) | United Arab Emirates | -Estimate the uptake of the recently introduced HPVV in the Emirate of Abu Dhabi.  - Explore barriers to uptake. | -A cross-sectional study through self-administered questionnaire.  -Duration: in 2011(questionnaire was distributed in the final year of medical students), on the day the MS visited the school  -Setting: School-based vaccine. | Size is 336 female students, from secondary girls’ schools with grades 11 and 12 in the district of Al Ain, age 15-20. | 27.5% had adequate knowledge about HPV.  35% stated that they had no information about HPV at all.  -The main sources of information were schools (39.8%), followed by media outlets (35%).  -Most preferred to take advice from healthcare providers (64%) and/or friends and family (59%).  83% supported a free vaccine for all.  53.3% received HPVV.  -Reasons for not taking the HPVV included side effects (33%), family refusal (21%), and costs (5%).  -Among those who hadn't received the HPV vaccine, 50% expressed an interest in receiving it in the future. | - Lack of knowledge about HPV infection & HPVV.  -Vaccine safety, efficacy & side effects.  -Vaccine cost. | -Vaccine provision as free of charge.  -Client’s awareness about CC, HPV & HPVV.  - Health care providers’ awareness about CC, HPV & HPVV.  . | -Raising awareness regarding HPV & related disease & HPVV. |
| 13 | (AlObaid et al., 2014) | Saudi Arabia | -Evaluate the prevalence and type distribution of HPV and document the awareness of HPV infection and health-related behaviour among Saudi and non-Saudi women attending routine examination. | -An observational, epidemiological cross-sectional study through self-administered questionnaire.  -Duration: 9 months, between April 2010 and December 2011.  -Setting: three large hospitals: King Fahd Medical City, King Faisal Specialist Hospital and Research Centre and King Abdulaziz Medical City-National Guard Health Affairs | -Size: 417 women, aged ≥15 years. | 32.2% had awareness about HPV.  89.9% demonstrated a willingness for vaccination. | - Lack of knowledge about HPV infection & HPVV. | -Willingness to receive HPVV. | - Initiation of HPV vaccination program.  - Raising awareness regarding HPV & related disease & HPVV |
| 14 | (Al Raisi et al., 2022) | Oman | -Assess the knowledge of and attitude toward CC and HPV among Omani women. | -cross-sectional structured questionnaire survey.  -Duration: 6 months, between September 2019 and February 2020.  -Setting: primary health centres across Oman. | Size: 805 women, aged ≥18 yrs. | 67.5% were aware about CC.  -The main source of information was social media (33%), followed by healthcare providers (16.9%).  -HPV infection as a risk factor was not well understood by the majority.  67.0% had never heard of HPV.  10% were aware of the HPV vaccine.  41.2% agreed to offer the vaccine to middle school-aged girls.  -Acceptance of vaccinating school children were notably related to education level, healthcare-related field of study, and employment status. | -Lack of knowledge about HPV infection & HPVV. | - Willingness to receive HPVV. | -Raising awareness regarding HPV & related disease & HPVV |
| 15 | (Alrajeh and Alshammari, 2020) | Saudi Arabia | -Assess the awareness, attitudes, and practice regarding HP and its vaccine among women attending primary care clinics in Riyadh. | -cross-sectional study through self-administered questionnaire.  -Duration: 12 months, between June 2014 and June 2015.  -Setting: primary care clinics of King Saud University Medical City (KSUMC) in Riyadh, Saudi Arabia. | Size: 326 women, aged 18–60 years. | 32.8% were aware that HPV is transmitted sexually.  21% were knowledgeable about HPV association with CC.  25% were aware about availability HPVV.  -The primary sources of information were doctors (5.6%), media (7.7%), and friends (24.5%).  30% displayed a positive inclination towards HPVV.  3.4% received the HPVV. | -Lack of knowledge about HPV infection & HPVV.  -Absence of national HPV vaccination program. | -. Willingness to receive HPVV. | - Initiation of HPV vaccination program. |
| 16 | (Al-Saadi et al., 2021) | Oman | -Assess knowledge of CC and its prevention among Omani women. | -cross-sectional study through self-administered questionnaire.  -Duration: 4 months, between November 2018 and February 2019.  -Setting: eight primary healthcare institutions in Al Buraimi Governorate, Oman. | Size: 791, aged 20–65 years. | 86.7% were aware of CC.  24.7% recognised the link between HPV & CC.  63.8% were unaware of HPVV availability.  73.8% showed willingness to receive the vaccine and supported its inclusion in the national immunisation program for girls in Oman. | - Lack of knowledge about HPV infection & HPVV. | - Willingness to receive HPVV. | -.Raising awareness regarding HPV & related disease & HPVV. |
| 17 | (Alsanafi et al., 2023) | Kuwait | -Investigate the willingness of female students at the University of Kuwait to get HPV vaccination and its possible association with general vaccine conspiracy beliefs (VCBs). | -A cross-sectional online survey study.  -Duration: 3 months, from September–November 2022.  -Setting: female students enrolled at Kuwait University (KU) | -Size: 611, aged <22 yrs & ≥22 y. | 55.3% were aware of HPV.  -Among those aware of HPV, 78.7% are aware of the HPV availability.  -HPVV uptake stood at 8.9%.  -higher vaccination rate related to older age (11.5% for individuals aged 22 years or older compared to 4.2% among students younger than 22 years).  69.8% expressed willingness to accept HPVV if provided for free.  -The main reasons for HPVV hesitancy were attributed to self-satisfaction and lack of confidence, followed by constraints.  The main sources of information were university courses (44.1%), followed by social media (40.5%) and the internet (38.8%).  The highest acceptance rates were among those relying on university courses (78.5%) and healthcare providers' information (77.7%). | -Lack of knowledge about HPV infection & HPVV.  -Vaccine cost.  . | -Client’s awareness about CC, HPV & HPVV.  -Health care providers’ awareness about CC, HPV & HPVV. | -Raising awareness regarding HPV & related disease & HPVV |
| 18 | (Al Sekri et al., 2021) | Oman | -Estimate the prevalence of cytological abnormalities in Papanicolaou (Pap) smears and related risk factors among Omani women. | -Cross-sectional study  -Duration: 7 months March to September 2019.  -Setting: gynaecology and family medicine clinics of Khoula Hospital and Sultan Qaboos University Hospital in Muscat. | -Size: 442 women, aged 21 and 65 years. | -Not a single woman among the participants had previously been administered the HPV vaccine. |  |  | - Initiation of HPV vaccination program. |
| 19 | (Al-Shaikh et al., 2014) | Saudi Arabia | -Data collected included socio-demographic data, knowledge of cervical cancer risk factors and clinical presentation, Pap smear, and HPVV acceptance. | -Cross-sectional study through self-administered questionnaire.  -Duration: 14 months, between December 2013 and February 2014.  -Setting: students in Health Colleges at Princess Nora Bint Abdul Rahman University, Riyadh, Saudi Arabia. | -Size: 1258 students in Health Colleges at Princess Nora Bint Abdul Rahman University, Riyadh.  -age 20.4±1.3 years. | 95.7% had low knowledge about CC.  -The acceptance of the vaccine was influenced by its cost. | -Vaccine safety, efficacy & side effects.  - Lack of knowledge about HPV infection & HPVV.  -Vaccine cost. | -Client’s awareness about CC, HPV & HPVV. | -Raising awareness regarding HPV & related disease & HPVV. |
| 20 | (Al-Shaikh et al., 2017) | Saudi Arabia | -Evaluate the effectiveness of the health education programme on the knowledge of HPV among female medical students. | -A quasi-experimental, pre-, and post-intervention study  -Duration: was conducted in 2014 at the Princess Nourah bint Abulrahman University, Riyadh.  -Setting: Princess Nourah bint Abdulrahman University (PNU), Riyadh, Saudi Arabia, and comprised female medical students. | -Size: 535, aged 20.3±1.3 years. | -After the intervention, there was a significant rise in knowledge levels. | -Vaccine safety, efficacy & side effects. | - Client’s awareness about CC, HPV & HPVV | - Initiation of HPV vaccination program |
| 21 | (Alshammari and Khan, 2022) | Saudi Arabia | -Determine university students’ knowledge, attitudes and perceptions regarding HPV and its vaccine. | -A cross-sectional study through self-administered questionnaire.  -Duration: 5 months, from January to May 2020.  -Setting: students enrolled at the University of Hail, Saudi Arabia. | -Size: 386.  -aged ≤20 - ≥30 | 33.7% were aware of HPV.  29.5% believe that HPV is transmitted sexually.  53.4% were not aware of health issues associated with HPV.  38.8% linked CC with HPV.  62.2% believed that no vaccine for HPV.  61.9% believed the vaccine does not reduce the risk of CC.  The participants showed a higher acceptance of taking HPVV if recommended by their physicians, followed by their friends. | -Lack of knowledge about HPV infection & HPVV.  -Absence of national HPV vaccination program. | -. Health care providers’ awareness about CC, HPV & HPVV. | - Raising awareness regarding HPV & related disease & HPVV. |
| 22 | (Al Shdefat et al., 2022a) | United Arab Emirates | -Determine Emirati men’s acceptability about the HPV vaccination, specifically whether they would use it themselves or allow their female relatives to use the vaccine. | -A cross-sectional study through questionnaire survey .  -Duration: in 2022.  -Setting: United Arab Emirates men. | -Size: 390  -age: not mentioned | 37% accepted HPVV.  46.7% were willing to endorse the HPVV for others.  Barriers for HPVV: 23.6% perceived no barriers,11.3% cited cultural unacceptability, 2.1% stated religious unacceptability, 5.4% linked it to women's lesser concern for their own health, 44% of the population remained unaware of the situation.  -Recommendations for the vaccine depended on its endorsement by:  13.3% through health programs in the media &  9.7% by doctors.  3.1% took the vaccine.  11% of the respondents' families had received the HPV vaccine. | -Cultural & religious belief.  -Vaccine safety, efficacy & side effects.  -Vaccine cost.  -Lack of knowledge about HPV infection & HPVV. | - Willingness to receive HPVV. | - Raising awareness regarding HPV & related disease & HPVV. |
| 23 | (Al Shdefat et al., 2022b) | United Arab Emirates | -Assess the knowledge and awareness of the HPVV among Emirati men. | -A quantitative cross-sectional survey.  -Duration: done in 2022  -Setting: not mentioned | Size: 390 Emirati men.  Age: not mentioned | 16.7% were familiar with HPV.  -The primary source of information for respondents is the Internet.  10.8% correctly identify HPV as a sexually transmitted disease.  17.2% agreed HPV can be prevented by the vaccine. | -Lack of knowledge about HPV infection & HPVV. |  | -Raising awareness regarding HPV & related disease & HPVV.  - Initiation of HPV vaccination program |
| 24 | (Alsous et al., 2021) | Jordan, Qatar, the United Arab Emirates (UAE), and Iraq. | -Investigate the knowledge and awareness about the HPVV among females in four Arab countries and their acceptance to receive the vaccine. | A cross-sectional study through Google forms.  Duration: done in 2021. | -Size: 2804  -397 were from Qatar.  -606 from the UAE.  Aged between 18 and 25 years old. | 15.4% (Qatar) & 30.7%(UAE) recognized that HPV is transmitted sexually.  24.7%(Qatar) & 42.1%(UAE) believed that HPV is resolved spontaneously.  - HPV is a cause for CC was identified by 28.5% (Qatar) and 51.2% (UAE).  -Awareness that the HPVV is effective against CC was observed in 11.8% (Qatar) and 26.9% (UAE).  -Awareness about the target group for vaccination was possessed by 20.2% (Qatar) and 16.3% (UAE).  35.5% (Qatar) and 48% (UAE) expressed a willingness to receive HPVV.  3.8% (Qatar) and 9.4% (UAE) would recommend the HPV vaccine for a child or adolescent aged 9–12.  19.9% (Qatar) and 35.3% (UAE) would recommend the HPV vaccine for a friend or relative. | - Lack of knowledge about HPV infection & HPVV  -Vaccine safety, efficacy & side effects. |  | -Raising awareness regarding HPV & related disease & HPVV. |
| 25 | (Sait, 2011) | Saudi Arabia | -Evaluate knowledge and practice of the Pap test, the role of HPV in the aetiology of CC, and attitudes regarding the HPV of female physicians in the Western Region of Saudi Arabia. | -A cross-sectional descriptive study using an interview with a structured questionnaire.  -Duration: 8 months, May, and December 2009.  -Setting: Department of Obstetrics & Gynecology, Faculty of Medicine, King Abdulaziz University Hospital, Jeddah, Kingdom of Saudi Arabia. | -Size: 200, aged 22-59 years. | 90% of the gynaecologist group (GD) and 60.5% of the non-gynaecologist group (NGD) were aware that HPV is a contributing factor to CC.  48.5% were informed about the HPVV.  -After being provided with information about the HPVV, 50% of physicians indicated they would recommend it to their patients. 47% would administer it to their daughters, and 42.5% were willing to receive it themselves. | - Lack of knowledge about HPV infection & HPVV. | - Clients’ awareness about CC, HPV & HPVV | Raising awareness regarding HPV & related disease & HPVV. |
| 26 | (Anfinan, 2019) | Saudi Arabia | -Appraise physicians’ preparedness to undertake the inherent actions and responsibilities, by evaluating their knowledge and opinions regarding HPV infection and vaccine. | -A cross-sectional study through self-administered questionnaire.  -Duration: between Jan 2017 and Nov 2018.  -Setting: 21 public canters from the five regions of Saudi Arabia. | -Size: 2000 physicians, aged 20- ≥60 yrs old. | 7.6% received the HPVV themselves.  41.2% expressed willingness to be vaccinated.  77.6% were open to vaccinating their own children.  69.6% supported the inclusion of the HPV vaccine in the local immunization program.  -Reasons for refusing the HPV vaccine among those who disagreed included:  Not perceiving themselves at risk of HPV infection (58.5%). Lack of knowledge about the vaccine (21.1%). Being sexually inactive (14.7%).  Concerns about the vaccine not being government-reimbursed (8.7%).  Fear of vaccine side effects (8.4%). | - Lack of knowledge about HPV infection & HPVV.  -Vaccine safety, efficacy & side effects. | -Health care providers awareness about CC, HPV & HPVV.  - Willingness to receive HPVV. | -Raising awareness regarding HPV & related disease & HPVV.  - Initiation of HPV vaccination program |
| 27 | (Azer et al., 2022) | Saudi Arabia | -Assess the knowledge of undergraduate medical and non-medical students about CC and HPVV. | -A cross-sectional study using Google forms.  -Duration: February 2020. | -Size: 172 students.  -Age: participants from the College of Medicine and the Arts and Business colleges in the final two years of their undergraduate course at the King Saud University. | 52.3% of medical students (MS) believed that the vaccine should be administered to both boys and girls, compared to 22.1% of non-medical students (NMS).  60.5% of (MS) expressed a willingness to receive the vaccine, whereas only 26.7% of (NMS) were open to vaccination.  -The main source of information: Medical courses for MS, while social media for NMS  -(MS) identified vaccine availability as the most common obstacle preventing them from receiving the vaccine. In contrast, (NMS)cited inadequate information as the main barrier. | - Lack of knowledge about HPV infection & HPVV. | - health care providers’ awareness about CC, HPV & HPVV. | -Raising awareness regarding HPV & related disease & HPVV. |
| 28 | (Darraj et al., 2022) | Saudi Arabia | -Evaluate participants’ knowledge, attitude, and acceptability concerning HPV and the HPVV in Jazan Province, Saudi Arabia. | A cross-sectional study through Google forms.  Duration: 3 months, between January and March 2022.  -Setting: Jazan Province, Saudi Arabia | Size: 569  Age: the mean age of participants was 26 years. | - The knowledge score was 20%.  54% agreed that males should be given the HPVV.  50% believed that HPV causes too few cancers among females.  -One-third believed that the vaccine would encourage riskier or sexual behaviour.  37% concerned about the efficacy.  45% were concerned about vaccine safety.  29% opposed HPVV for moral or religious reasons.  53% were interested in the HPV vaccine for males. | - Lack of knowledge about HPV infection & HPVV.  -misconception that receiving HPVV may encourage risky sexual behaviour.  -Vaccine safety, efficacy & side effects.  - Cultural & religious beliefs.  -vaccine cost |  | - Raising awareness regarding HPV & related disease & HPVV.  -Initiation of HPV vaccination program |
| 29 | (Dhaher, 2019) | Saudi Arabia | - Measure women’s knowledge, attitudes and practices about CC and the Pap smear test in the southern region of Saudi Arabia .  -Assess the findings in relation with women’s demographics | A cross sectional survey through self-administered questionnaire.  Duration: 2 months, from March to April 2017.  -Setting: Armed Forces Hospital Southern Region Obstetrics and Gynecology Clinic. | Size: 255, aged 15 - 65 years | 43% were aware of CC.  -The main source of information was social media.  -Low awareness about HPVV.  -Only one woman received the vaccine in the United States. | - Lack of knowledge about HPV infection & HPVV.  -Absence of HPV vaccination program. |  | - Raising awareness regarding HPV & related disease & HPVV. |
| 30 | (Elgzar et al., 2022) | Saudi Arabia | -Investigate the Saudi women's intention and self-efficacy for Pap Smear Screening and HPVV in Najran city, KSA. | A descriptive cross-sectional study through a social media questionnaire.  -Duration: over nearly four months (from October 2021 till the end of January 2022) | -Size: 1085 Saudi women, aged ≥18 yrs. | 62.5% expressed a strong intention to receive the HPVV.  -Factors associated with higher willingness to receive the HPVV included being of reproductive age and having a high gravidity and parity. | - Cultural & religious beliefs.  - Lack of knowledge about HPV infection & HPVV. |  | - Raising awareness regarding HPV & related disease & HPVV.  - Initiation of HPV vaccination program |
| 31 | (Farsi et al., 2020) | Saudi Arabia | -Assess knowledge about HPV, vaccination, and HPV-related oropharyngeal cancer (OPC).  -Evaluate HPV vaccine acceptability among a sample of undergraduate dental students. | -A cross-sectional study through a self-administered questionnaire.  -Duration: 8 months, between March and October 2018  -Setting: dental schools in Jeddah, Saudi Arabia | Size: 500 students  Age: dental students enrolled during academic year 2018-2019 in third or fourth year | 62% were aware about HPV infections.  57% were familiar with HPVV.  -Better knowledge associated with higher willingness to receive the HPVV.  -Those who received HBV vaccine were more willing to take HPVV.  4% of males and 12% of females, received the HPV vaccine.  -Among those who declined the vaccine, 51% cited a lack of knowledge as their primary reason for refusal. | - Lack of knowledge about HPV infection & HPVV. | Health care providers’ awareness about CC, HPV & HPVV.  . | - Raising awareness regarding HPV & related disease & HPVV. |
| 32 | (Farsi et al., 2021) | Saudi Arabia | -Assess knowledge about HPV and the vaccine, as well as its acceptability, among third- and fourth-year male medical students in Jeddah, Saudi Arabia. | -cross-sectional study through self-administered questionnaire.  -duration:10 months, from February to December 2018. | Size: 517 participants.  Age: mean ± SD 21 ± 1.4. | 74% were aware about HPV.  42% were familiar with HPV vaccine.  48.9%, showed willingness in getting the HPVV.  -Those who received the HBV vaccine were more willing to get the HPV vaccine. | - Lack of knowledge about HPV infection & HPVV. | - Willingness to receive HPVV. | - Raising awareness regarding HPV & related disease & HPVV.  - Initiation of HPV vaccination program |
| 33 | (Gari et al., 2012) | Saudi Arabia. | -Assess the knowledge and the attitude toward the CC screening (the Pap smear) among women in Saudi Arabia. | A cross-sectional study. Self-administered questionnaires to schoolteachers, relatives and friends  Duration: 2 months, from November to December 2011. | Size: 1023 women, aged 16 to 45 years old. | 84.6% had no knowledge of HPV.  82.2% were unaware that HPV could cause CC.  20.8% were aware of HPVV availability.  - The main source of information was media.  1.4% received the vaccine.  63.3% willing to receive the vaccine.  -Reasons for not receiving the vaccine: a lack of knowledge (43.6%), fear of side effects (29.7%), lack of trust (11.9%), thinking it's not important (8.2%), costs (2.9%), and other reasons (4%).  63.2% recommend the vaccine to others. | -Lack of knowledge about HPV infection & HPVV.  -Vaccine safety, efficacy & side effects.  -Vaccine cost. | - Willingness to receive HPVV | -Raising awareness regarding HPV & related disease & HPVV. |
| 34 | (Husain et al., 2019) | Bahrain | -Determine the level of awareness of HPV infection and to assess attitudes towards receiving the vaccine among men and women in Bahrain. | A cross-sectional study through interview-based questionnaire.  Duration: one month, in February 2018. | Size: 408 PHC attendees, including 268 women and 140 men, aged 18–65 years. | 13.5% were aware about HPV.  76% were willing to receive the vaccine if recommended.  84.8% believed that both genders should receive the vaccine.  48.5% worried about side effects.  -83.6% worried about effectiveness. | -Lack of knowledge about HPV infection & HPVV.  -Vaccine safety, efficacy & side effects. | - Willingness to receive HPVV | - Initiation of HPV vaccination program. |
| 35 | (Ibrahim et al., 2022) | Saudi Arabia | -Explore Saudi women's health beliefs and associated factors regarding CC prevention in Najran city. | -A cross-sectional study through an online survey  -Duration: 4 months, from June to September 2021 | -Size: 1085 women aged 20 to 60 years. | 99% did not receive the HPVV.  -Factors such as residing in rural areas, lower education levels, inadequate monthly income, and younger age were negatively associated with CC prevention and health beliefs. | -Lack of knowledge about HPV infection & HPVV. |  | Raising awareness regarding HPV & related disease & HPVV. |
| 36 | (Jassim et al., 2018) | Bahrain | -Explore the knowledge, attitudes, and practices of women attending primary care health centres for cervical cancer screening. | -A cross-sectional study through questionnaire and face-to-face interviews by trained Arabic-speaking female interviewers  -Duration: 3 months, between December 2015 and February 2016  -Setting: women attending primary health care centres in Bahrain. | -Size: 300 women, the mean age is 37.24 ± 11.89 years. | 3.7%, had heard about HPVV.  81.8% were willing to receive the vaccine.  90.9% were willing to vaccinate their children. | -Lack of knowledge about HPV infection & HPVV. | - Willingness to receive HPVV. | - Raising awareness regarding HPV & related disease & HPVV.  - Initiation of HPV vaccination program |
| 37 | (Jradi and Bawazir, 2019) | Saudi  Arabia | -Examine the awareness of HPV and women’s attitudes toward the HPVV. | -A qualitative study through face-to-face interviews.  -Duration: 3 months, between September and November 2016 | -Size:77 women + 58 female health care providers, aged 18 to 45 years old. | - Low awareness about CC, HPV, and the HPVV.  -Cultural concerns about screening and vaccination for sexually transmitted infections.  30% of healthcare providers, excluding physicians, were not knowledgeable about preventive measures.  63.3% of healthcare providers did not perform any screening for CC. | - Lack of knowledge about HPV infection & HPVV.  -Cultural & religious beliefs. |  | - Raising awareness regarding HPV & related disease & HPVV. |
| 38 | (Hendaus et al., 2021) | Qatar | -Delineate parental attitude regarding HPV in Qatar. | -A cross-sectional study through a questionnaire.  -Duration: 12 months, from April 1, 2019 to March 30, 2020.  -Setting: paediatric inpatient and outpatient departments of Sidra Medicine. | -Size: 334, aged between 20 and 39 years. | 60%, were unaware that HPV can lead to CC & other cancers.  77% were willing to vaccinate their children.  4% admitted that a paediatrician had ever mentioned the existence of HPVV.  54% preferred to take the advice about the vaccine from health care providers.  20% had doubts about the HPVV. | -Misconception that receiving HPVV may encourage risky sexual behaviours.  -Vaccine safety, efficacy & side effects. | - Willingness to receive HPVV.  -Health care providers’ awareness about CC, HPV & HPVV. | - Initiation of HPV vaccination program. |
| 39 | (Ortashi et al., 2014) | -United Arab Emirates | -Assess the knowledge of women regarding HPV infection and vaccine in UAE. | -A cross-sectional study through questionnaire by face-to-face interviews was conducted among eligible women  -Duration: 7 months, April 2012 to October 2012.  -Setting: Homes, shopping malls, workplaces, and community centres. | -Size: 640 women, aged 18-50 years | 37% had the knowledge about HPVV.  80% were willing to receive the vaccine, and 87% would recommend it to relatives or friends.  69% had a positive opinion about the vaccine.  17% & 1% worried about cultural & religious issue respectively.  -HPVV uptake could be enhanced by vaccine safety and doctor's recommendations, cited by 36% each.  6% received HPVV.  -Being a national and having a more educated husband were associated with a positive attitude towards the HPVV. | - Lack of knowledge about HPV infection & HPVV.  -Vaccine safety, efficacy & side effects.  -Cultural & religious beliefs. | - Willingness to receive HPVV.  -Vaccine provision free of charge.  -Health care awareness about CC, HPV & HPVV. |  |
| 40 | (Ortashi et al., 2013) | -United Arab Emirates | -Assess the knowledge about and acceptability of HPVV among male university students in UAE. | -A cross-sectional quantitative survey. self-administered questionnaire. Male Students from all colleges of the UAE University were asked to participate.  -Duration: 3 months, between, June and August 2012. | -Size: 356 male university students  Age: university students. | -Most students were not aware of HPV.  46% showed willingness to receive the HPVV.  -Factors enhanced vaccine acceptance: (68%) trust in vaccine safety &(50%) protection against CC.  85% worried about side effects.  27% knew the link between HPV infection and CC. | - Lack of knowledge about HPV infection & HPVV.  -Vaccine safety, efficacy & side effects.  -Cultural & religious beliefs. | -Clients’ awareness about CC, HPV & HPVV. |  |
| 41 | (Ortashi et al., 2012) | -United Arab Emirates. | -Assess the knowledge, attitude, and practice of school nurses in the Emirate of Abu Dhabi about HPV infection and the vaccine. | -A quantitative study. questionnaire, data were collected by face-to-face interview.  -Duration: 3 months, from June to August 2012 | -Size: 125 nurses, aged < 30 to >40 | 97% were aware of HPV & HPVV.  71% had positive attitude toward HPVV.  45% admitted that Cultural unacceptability was a barrier.  58% reported either administering the vaccine to schoolgirls or receiving it themselves.  95% had no side effects.  -HPVV uptake among schoolgirls has risen to 80%. | - Cultural & religious beliefs. | -Health care providers’ awareness about CC, HPV & HPVV. | - Initiation of HPV vaccination program. |
| 42 | (Rezqalla et al., 2021) | -Kuwait | -Assess the prevalence of  i)unawareness of HPV infection’s causal role in CC.  ii) unawareness of HPVV availability.  iii) examined the sociodemographic variables associated with both outcome variables. | -A cross-sectional study. -A self-administered questionnaire was conducted among female schoolteachers employed in public and private sector schools in Kuwait.  -Duration: January 2018. | -Size: 1341 female schoolteachers, aged from 21 to 60 years | 60% were unaware about the link between HPV & CC.  88% were unaware about HPVV availability.  1.9% received the vaccine. | - Lack of knowledge about HPV infection & HPVV. |  | -Raising awareness regarding HPV & related disease & HPVV. |
| 43 | (Sait, 2009) | Saudi Arabia | -Assess the knowledge, attitude, and practices related to CC screening, and its underlying aetiology and preventive measures among women in the Kingdom of Saudi Arabia. | -A cross-sectional study A self-administered questionnaire + direct interview: these questionnaires were sent out to schoolteachers, relatives, friends, as well as through direct interview  -Duration: 3 months, from January to March 2008. | -Size: 500.  -Age: median age was 42 | 14.4% were aware that HPV is a cause CC.  9.8% were aware of HPVV. | - Lack of knowledge about HPV infection & HPVV. |  | -Raising awareness regarding HPV & related disease & HPVV. |
| 44 | (Saqer et al., 2017) | -United Arab Emirates | -Assess the knowledge and attitudes of parents in Sharjah towards HPV and whether they would vaccinate their daughters. | -A quantitative, observational cross-sectional study. -A self-administered questionnaire was conducted in public venues in Sharjah  -Duration: a two-month period, from February to April 2015. | -Size: 400. -Age: parents of any age, any nationality and who have daughters of any age. | 78.3% were aware of CC.  41.3% had knowledge of HPV.  36.5% were familiar with the HPVV.  76.6% showed their willingness to vaccinate their daughters, and this raised to 92.9% if the Ministry of Health recommended the vaccine. | - Lack of knowledge about HPV infection & HPVV. | -Willingness to receive HPVV.  -Health care awareness about CC, HPV & HPVV. |  |
| 45 | (Sundaram et al., 2021) | -United Arab Emirates | -Assess the awareness and opinions of female expatriate students of a private university regarding HPV, vaccination, and CC. | -A cross-sectional study. -A survey questionnaire was distributed among female expatriate students at a private university  Duration: done in 2021. | -Size: 269, aged from 18 to 26 years old | 6% had participated in awareness programs.  11% mentioned having read informational leaflets.  78% were unaware of HPV.  -Less than 40% believed that CC is preventable.  20% expressed disinterest in getting vaccinated.  5% received the vaccine. | - Lack of knowledge about HPV infection & HPVV.  -Vaccine cost.  -Vaccine safety, efficacy & side effects. |  | -Raising awareness regarding HPV & related disease & HPVV.  - Initiation of HPV vaccination programme. |
| 46 | (Tehsin et al., 2021) | Saudi Arabia | -Assess the awareness of CC among the King Faisal University community visiting the gynaecology clinic, Al Ahsa, Saudi Arabia. | -A cross-sectional study. Questionnaire  -Duration: two months. | -Size: 385 females, aged above 18 years | 73.2% were aware of CC.  -A significant number of Saudi nationals were aware of HPVV. |  | -Clients’ awareness about CC, HPV & HPVV | -Raising awareness regarding HPV & related disease & HPVV. |
| 47 | (Zahid et al., 2022) | Saudi Arabia | -Assess knowledge and practices related to CC among women in the Al Madinah Province in Saudi Arabia. | -A cross-sectional study. -online self-reported questionnaire among women in the Al Madinah Province in Saudi Arabia.  -Duration: done in 2022. | -Size: 1489, aged 18 years or older. | -Low awareness level about CC.  34% were aware that a sexually transmitted virus is a risk factor for CC.  12.6% were aware of HPVV availability. | -. Lack of knowledge about HPV infection & HPVV. |  | - Raising awareness regarding HPV & related disease & HPVV. |
| 48 | (Mahmoud et al., 2024) | The Gulf Cooperation Council (GCC) countries | to assess HPV vaccination and Pap test uptake, awareness, and barriers among young adults in the GCC countries. | -cross-sectional study -online cross-sectional survey  -from January to April 2024. | -Size: 831 participants. -Age: 18–39 years. | -The vaccination rate was higher among UAE participants followed by Qatar participants.  -Awareness about HPV infections, vaccinations, and recommendations to others was higher among UAE & KSA participants. | -Lack of knowledge about HPV infection & HPVV.  -Absence of the HPV vaccination program.  -vaccine cost.  -Vaccine safety, efficacy & side effects | -Willingness to receive HPVV.  -Health care awareness about CC, HPV & HPVV. | - |
| 49 | (Cheema et al., 2024) | Qatar | -To evaluate students’ knowledge, perceptions, and attitudes towards HPV infection | -cross-sectional study -A paper-based, self-administered, English questionnaire  -Between February and September 2022 | Size: 400 students from seven universities located in Education City, Doha, Qatar. Age: ≥ 18 | -Most of the students showed awareness about HPV infections and the vaccine.  6.3% were vaccinated. | Lack of knowledge about HPV infection & HPVV.  -Absence of HPV vaccination program.  -vaccine cost. | -willingness to receive the vaccine.  -Health care awareness about CC, HPV & HPVV. |  |
| 50 | (Alosaimi et al., 2024) | Saudi Arabia | To assess the knowledge and attitudes of physicians toward cervical cancer, HPV, and the HPV vaccine, and unravel factors that influence recommending the vaccine | Cross-sectional study between July 2023 and October 2023  Setting: Physicians working in Second Health Cluster in Riyadh, KSA | Size: 128  Age: median age is 30 (26-38) | (64 out of 128) correctly answered questions regarding cervical cancer, HPV, HPVV, indicating good knowledge.  77% learned about the HPVV at university, 58% from the media, and 63% from reading papers and attending conferences  69 out of 128 participants (54%) had a positive attitude towards HPV vaccination | -fear of vaccine side effects (15%)  -hesitancy to encourage students/daughters to take the vaccine (18.5%)  -Discomfort with sexual education (38%)  -Poor knowledge of the asymptomatic nature of HPV infection | -Physicians personal reading (91%)  -Recommendations from colleagues (88%)  -Government directives (87%)  -Longer clinical exposure (>4yrs) and institutional awareness  -Physicians in vaccine-relayed medical specialities 5 times more likely to have good knowledge | -Design interventions targeting specific demographic and professional groups to improve knowledge and promote positive attitudes  -Improve awareness of physicians and their attitudes towards vaccination  -Revisit the medical curriculum in Saudi Arabian health colleges |
| 51 | (Albayat et al., 2024) | Qatar | To assess the knowledge, attitudes, and practices among physicians working in Qatar regarding HPV infection and prevention using vaccines, and barriers to advising HPV vaccination to patients | Cross-sectional study using quantitative data collection  Duration: Oct 2021 to Sept 2022  Setting: Online-based survey among physicians in healthcare settings of Qatar | Sample size: 557 physicians  Mean age 44.6 (20-70 range) | 83.7% of physicians had sufficient knowledge regarding HPV infection and HPV vaccine.  Majority knew HPV infection could be asymptomatic (89.4%) and at least one symptom (96.1%).  only 69.1% knew that HPVV were available in Qatar  Only 33.9% knew correct doses for the HPV vaccine  4.8% reported having received the HPV vaccine themselves  More than one-third of the physicians were not interested in recommending the HPV vaccine  Majority knew HPV vaccination should be given before sexual activity (84.0%) and that both males and females could be vaccinated (79.7%).  77% believed the HPV vaccine would substantially decrease the chances of HPV infection and related cancers | Lack of awareness HPV and CC (61.6%).  Doubts regarding vaccine efficacy (32.5%)  Fear regarding vaccine safety (26.9%)  Concern that HPVV may encourage risky sexual behaviour (26.8%)  Perceived low-risk in the population (23.3%)  Cost (24.6%)  Physicians were not commonly discussing sexual health with their clients (44.5% rarely, 33.1% sometimes)  More than one-third of physicians were not interested in recommending the HPV vaccine.  Many physicians not willing to vaccinate their own adolescent daughters (16.9% hesitant), primarily due to the belief that "their daughters are not sexually active" (41.0%)  Other reasons included no need, concerns about safety/effectiveness, spouse/family opposition, or no specific reason.  Disinterest in receiving the HPVV themselves (only 4.8% had received it)  Misconception that HPV infection is curable (34.5%) | Majority believed cervical cancer is a major concern for women (91.9%).  Non-Qatari physicians had significantly higher sufficient knowledge than Qatari physicians (84.5% vs. 72.9%).  Paediatricians had a greater proportion of sufficient knowledge.  Physicians with sufficient knowledge were more likely to discuss sexual health and recommend HPV vaccines.  Christians were 4 times more likely to recommend than Muslims, and Hindu/Jain/Buddhist were 3.5 times more likely.  Most physicians were very likely to recommend the HPV vaccine if offered free of charge (73.6% willing to vaccinate their daughters, 68.6% would recommend if free). | Targeted education and tailored advocacy activities are required to address gaps in knowledge and attitudes of healthcare providers.  Empower healthcare providers to address perceived barriers and misunderstandings through suitable approaches and effective communication.  Organise awareness classes/workshops for physicians to equip them to respond to community queries and rumors.  Address physician disinterest in HPV vaccination to avoid possible vaccination hesitancy.  Setting appropriate strategies and organising interventions and services are mandated for the enhancement of a comprehensive cervical cancer control programme in Qatar. |
| 52 | (Abu Sanad et al., 2024) | Saudi Arabia | To evaluate the attitude, perception, and understanding of cervical cancer, its risk factors, the HPV infection, and vaccinations among women in various regions of Saudi Arabia | Cross-sectional study  Duration: September 2022 to January 2023  Setting: Online-based questionnaire | Sample size: 2539 women  Age: 41% were 18–25 years old, and 49.4% were 31 years and above | (30.5%) knew that the HPVV prevents CC  84% had inadequate awareness of CC and HPV  Only 2% (50 out of 2539) had received the HPVV  Low utilisation of Pap smear (20%)  Two-thirds of participants did not know HPVV prevent CC, and benefits.  Almost half of the women did not know the appropriate age for vaccination. | Majority had inadequate CC awareness (median awareness score of five out of 15, or 33.3%)  "I do not know its importance" was the most common reason for not doing a Pap smear (32.4%)  Cultural and religious beliefs. Poor access to healthcare and deficiency of nationwide screening programmes. Misconceptions about Pap smears. Lack of knowledge on how often a Pap smear should be taken (60% did not know)  Extreme shyness (6.9%) and belief that the pap smear test was ineffective (5.6%).  Concerns about social media as a source of information, as it may not always be accurate.  The majority (60.8%) did not know whether the vaccine is free or not. | Eastern region residents, medical field occupation, and income higher than 20K SR per month were associated with higher awareness levels.  Age, family income, and educational level were significant predictors for performing a Pap smear.  Students and professionals in the medical field had a substantially higher level of awareness. | Better advocacy and implementation of nationwide campaigns are needed to further utilise early detection and preventive procedures.  Integrating knowledge and awareness educational programmes and advocating for HPVV are crucial to reducing HPV infection and CC mortality.  Educational programmes should not exclude women with a high level of education and socioeconomic status.  Raise awareness among women about cervical cancer screening through educational campaigns throughout the Kingdom to encourage early testing. |
